# Genetic Diversity and Pathogenicity of *Botryosphaeriaceae* Species Associated with Symptomatic Citrus Plants in Europe

**DOI:** 10.3390/plants10030492

**Published:** 2021-03-05

**Authors:** Jadson Diogo Pereira Bezerra, Pedro Wilhelm Crous, Dalia Aiello, Maria Lodovica Gullino, Giancarlo Polizzi, Vladimiro Guarnaccia

**Affiliations:** 1 Setor de Micologia, Departamento de Biociências e Tecnologia, Instituto de Patologia Tropical e Saúde Pública (IPTSP), Universidade Federal de Goiás (UFG), Rua 235, s/n, Setor Universitário, Goiânia 74605-050, Brazil; jadsondpb@gmail.com; 2 Westerdijk Fungal Biodiversity Institute, Uppsalalaan 8, 3584 CT Utrecht, The Netherlands; p.crous@wi.knaw.nl; 3 Dipartimento di Agricoltura, Alimentazione e Ambiente, sez. Patologia Vegetale, University of Catania, Via S. Sofia 100, 95123 Catania, Italy; dalia.aiello@unict.it (D.A.); gpolizzi@unict.it (G.P.); 4 Centre for Innovation in the Agro-Environmental Sector, AGROINNOVA, University of Torino, Largo Braccini 2, 10095 Grugliasco, Italy; marialodovica.gullino@unito.it; 5 Department of Agricultural, Forest and Food Sciences (DISAFA), University of Torino, Largo Braccini 2, 10095 Grugliasco, Italy

**Keywords:** *Diplodia*, *Dothiorella*, *Lasiodiplodia*, *Neofusicoccum*, pathogenic fungi, phylogeny

## Abstract

This study represents the first survey studying the occurrence, genetic diversity, and pathogenicity of Botryosphaeriaceae species associated with symptomatic citrus species in citrus-production areas in five European countries. Based on morphological features and phylogenetic analyses of internal transcribed spacer (ITS) of nuclear ribosomal DNA (nrDNA), translation elongation factor 1-alpha (*TEF1*) and β-tubulin (*TUB2*) genes, nine species were identified as belonging to the genera *Diplodia*, *Dothiorella*, *Lasiodiplodia,* and *Neofusicoccum*. Isolates of *Neofusicoccum parvum* and *Diplodia pseudoseriata* were the most frequently detected, while *Dothiorella viticola* had the widest distribution, occurring in four of the five countries sampled. Representative isolates of the nine Botryosphaeriaceae species used in the pathogenicity tests caused similar symptoms to those observed in nature. Isolates assayed were all re-isolated, thereby fulfilling Koch’s postulates. Isolates of *Diplodia pseudoseriata* and *Diplodia olivarum* are recorded for the first time on citrus and all species found in our study, except *N. parvum*, are reported for the first time on citrus in Europe.

## 1. Introduction

Citrus production represents one of the most important fruit industries worldwide in terms of total yield. Greece, Italy, Portugal, and Spain are the most important European producers of citrus fruit [1]. In 2019, nearly 11 million tons of citrus was produced in Europe on approximately 515,000 ha [2]. Most canker diseases of citrus, as well as further fruit-tree crops, are caused by a broad range of fungal species that infect the wood mainly through winter pruning wounds and a subsequent colonization of vascular tissues [3]. Several abiotic and biotic factors are considered responsible for rots and gumming on the trunk and main branches in citrus. Frost damage, sunscald, or water distribution can promote the infection of numerous ascomycetes and basidiomycetes [4]. Several fungal infections involving twigs, branches and trunks of citrus caused by *Colletotrichum* and *Diaporthe* species were reported in different continents [5,6,7,8,9]. Guarnaccia and Crous [10] reported serious cankers developing in woody tissues of lemon trees caused by *Diaporthe* spp., often with a gummose exudate, causing serious blight and dieback. Canker diseases of citrus are also caused by other fungal genera such as *Fusarium* and *Neocosmospora* [11], *Peroneutypa* [12,13], and *Phaeoacremonium* [14]. Recently, significant attention has been dedicated to revising species and genera of Botryosphaeriaceae, which encompass species with a cosmopolitan distribution that are able to cause diseases of numerous plant species worldwide [15,16].

Botryosphaeriaceae (Botryosphaeriales) include several species reported as endophytes, latent, and woody plant pathogens on a broad range of host [15,16,17]. This family has undergone significant revision after the adoption of molecular tools to resolve its taxonomy [15,16,18,19,20,21,22,23]. Recently, the taxonomy of Botryosphaeriaceae (and other families in Botryosphaeriales) has been reviewed by Phillips et al. [23] based on morphology of the sexual morphs, phylogenetic relationships on internal transcribed spacer (ITS) and 28S large subunit (LSU) of nuclear ribosomal DNA (nrDNA) sequences and evolutionary divergence times. The authors highlighted the main findings made by Yang et al. [16] who included new families, genera, and species in Botryosphaeriales based on morphology and multi-marker phylogenetic analyses of a large collection of isolates. Currently, six families are accepted in Botryosphaeriales and 22 genera have been included in Botryosphaeriaceae [23,24,25]. 

The most common symptoms observed in association with species of Botryosphaeriaceae are twig, branch and trunk cankers, die-back, collar rot, root cankers, gummosis, decline and, in severe cases, plant death [15,17]. Plant infections mainly occur through natural openings or wounds, but these fungal species could also survive in latency. This ability could lead to their spread worldwide through asymptomatic plant material, seedlings and fruit, frequently circumventing the adopted quarantine measures [22]. Moreover, stress and non-optimal plant growth conditions consistently induce the expression of diseases associated with Botryosphaeriaceae species. Thus, global warming could increase plant stress and induce favourable conditions for the development of Botryosphaeriaceae diseases [17,26,27]. Species within the Botryosphaeriaceae represent a serious threat to different crops including major fruit, berry fruit and nut crops cultivated in sub-tropical, tropical, or temperate areas [22,28,29,30].

Several species of *Diplodia* (*Di*.), *Dothiorella* (*Do*.), *Lasiodiplodia*, *Neofusicoccum,* and *Neoscytalidium* (*Ne*.) have been previously reported to affect *Citrus* species [13,31,32,33]. For example, *Ne. dimidiatum* has been reported causing citrus branch canker in California [13] and Italy [32]; *Do*. *viticola*, *L*. *citricola*, *L. theobromae,* and *Ne. dimidiatum* have been described in association with branch and trunk dieback of citrus trees in Iran [14,34] and *Dothiorella* spp. have been detected as causal agents of citrus gummosis in Tunisia [35]. Moreover, *Di. seriata*, *Di. mutila*, *Do. viticola*, *L. mediterranea* and *L. mitidjana,* have been recovered from symptomatic citrus trees in Algeria [33].

Considering the important economic value of *Citrus* spp., a large survey of Botryosphaeriaceae affecting plants cultivated in the major citrus production areas of Europe was considered imperative. Identification in light of modern taxonomic concepts via morphological characterization and multi-marker DNA sequence data was necessary to adopt efficient control strategies against the pathogens that could affect these crops. Thus, several surveys have been conducted in Greece, Italy, Portugal, Spain, and Malta during 2015 and 2016. In particular, the aims of this study were to (1) conduct extensive surveys for sampling symptomatic plant materials; (2) obtain a broad collection of Botryosphaeriaceae isolates; (3) subject those isolates to DNA multi-marker sequence analyses combined with morphological characterization, and (4) evaluate the pathogenicity of the isolated species to citrus plants.

## 2. Results

### 2.1. Field Sampling and Fungal Isolation

**Figure 1:**
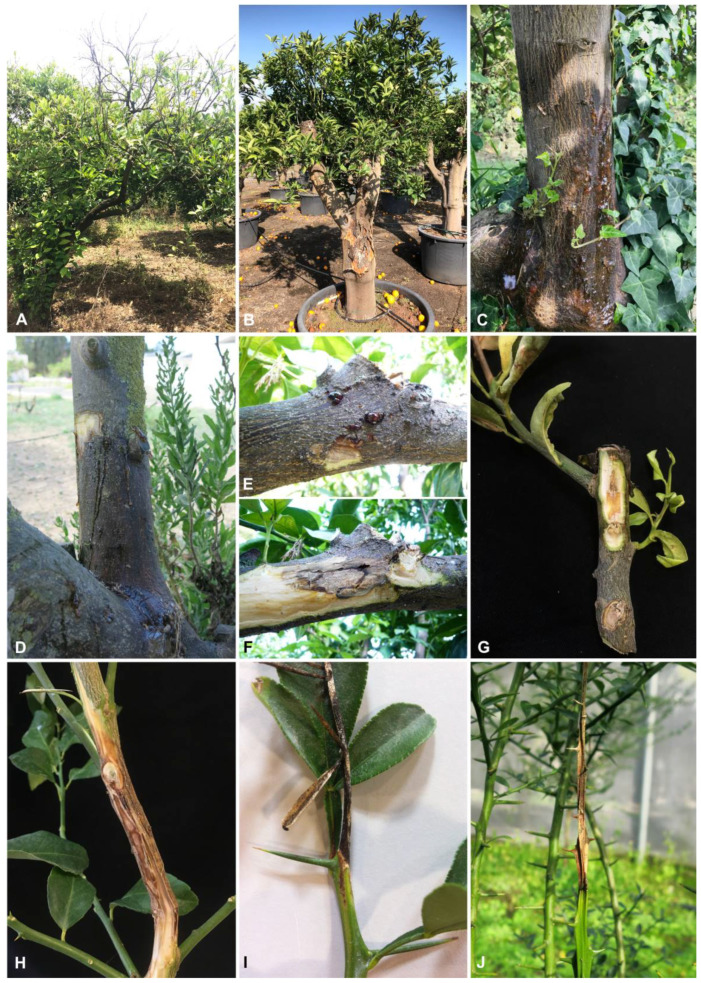
Symptoms on citrus tissues with associated Botryosphaeriacae species. (**A**) Branch decline in commercial lemon orchard. (**B**) Trunk canker and bark cracking of *C. sinensis.* (**C**,**D**) Trunk and branch canker with gummosis of *C*. *sinensis* plants. (**E**,**F**) External cracking with gummosis and internal wood discoloration of the same affected branch of *C. reticulata* plant. (**G**,**H**) Internal wood discoloration and branch blight of *C. limon*. (**I**) Twig dieback of young *C. sinensis* × *P. trifoliata* and *M. australasica* (**J**) plants.

In this study, the sampling focused on symptomatic plants of *Citrus limon*, *C. reticulata*, *C. sinensis*, *C. sinensis* × *Poncirus trifoliata,* and *Microcitrus australasica*. Samples were collected in 19 orchards (Table 1). Citrus trees showed various external disease symptoms, including partial or complete yellowing, wilting leaves and twigs, and dieback of branch tips, but also defoliation and branch decline. Canker and cracking of the bark associated with gummose exudate occurred on trunks and branches. Internal observation of infected branches revealed black to brown wood discoloration in cross-sections, wedge-shaped necrosis or irregular wood discoloration. Twigs were wilted and occasionally presenting sporocarps (Figure 1). Symptoms were detected in all the orchards and regions investigated. A total of 63 fungal isolates were collected and were found to be characterized by dark green to grey, fast-growing mycelium on MEA. Moreover, the isolates produced pycnidia on pine needles within 40 days, containing pigmented or hyaline conidia. According to these characteristics, the fungal isolates were classified as Botryosphaeriaceae spp. based on comparison with the previous generic descriptions [15]. Among the collected isolates, 18 were obtained from trunk cankers, 10 were associated with branch infections, and 35 from twig dieback (Table 2). 

### 2.2. Phylogenetic Analyses

**Figure 2:**
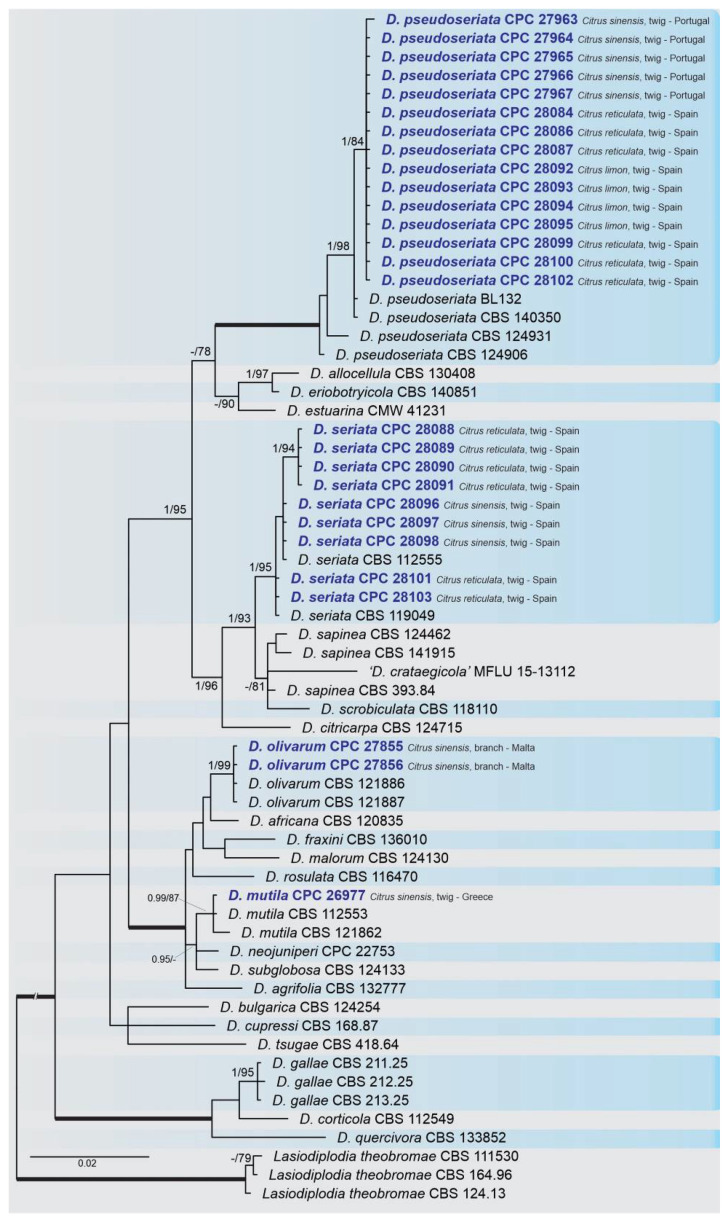
Bayesian inference analysis of *Diplodia* species using ITS rDNA, *TEF1* and *TUB2* sequences. Isolates obtained in this study are in bold and blue. Bayesian posterior probability (BPP) and maximum likelihood-bootstrap (ML-BS) values equal or greater than 0.95 and 70%, respectively, are shown near nodes. Thickened branches represent clades with ML-BS = 100% and a BPP = 1.0. The tree was rooted to *L. theobormae* (CBS 111530, CBS 164.96 and CBS 124.13).

**Figure 3:**
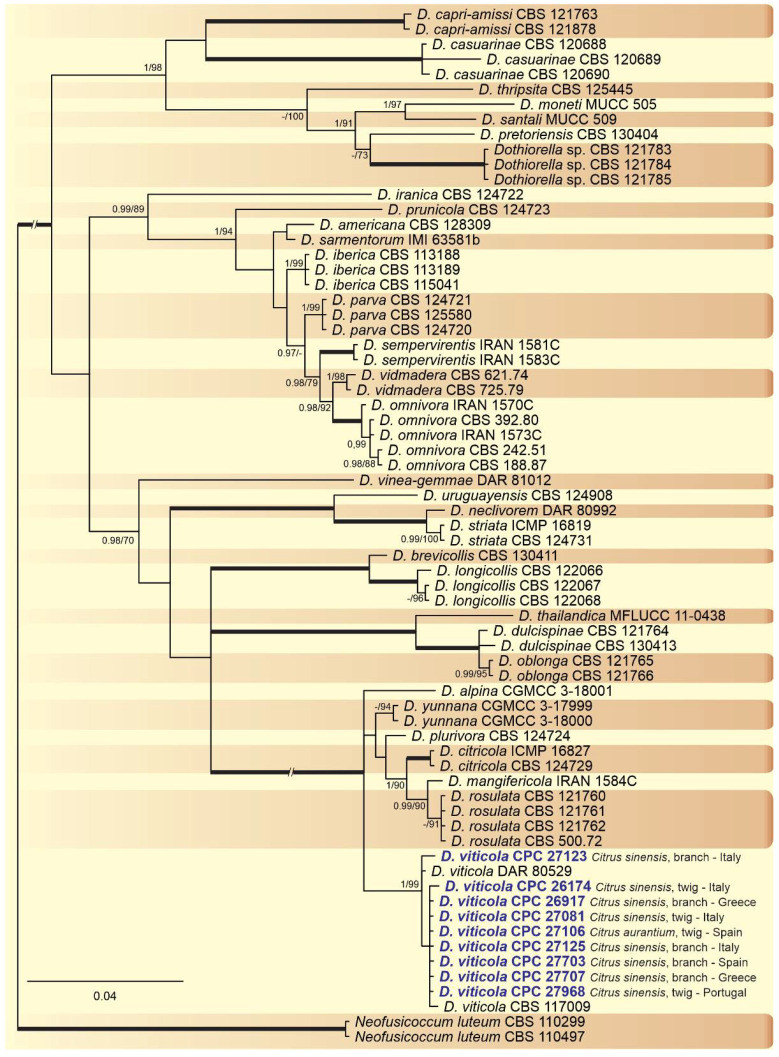
Bayesian inference analysis of *Dothiorella* species using ITS rDNA, *TEF1*, and *TUB2* sequences. Isolates obtained in this study are in bold and blue. Bayesian posterior probability (BPP) and ML bootstrap (ML-BS) values equal or greater than 0.95 and 70%, respectively, are shown near nodes. Thickened branches represent clades with ML-BS = 100% and a BPP = 1.0. The tree was rooted to *N. luteum* (CBS 110299 and CBS 110497).

**Figure 4:**
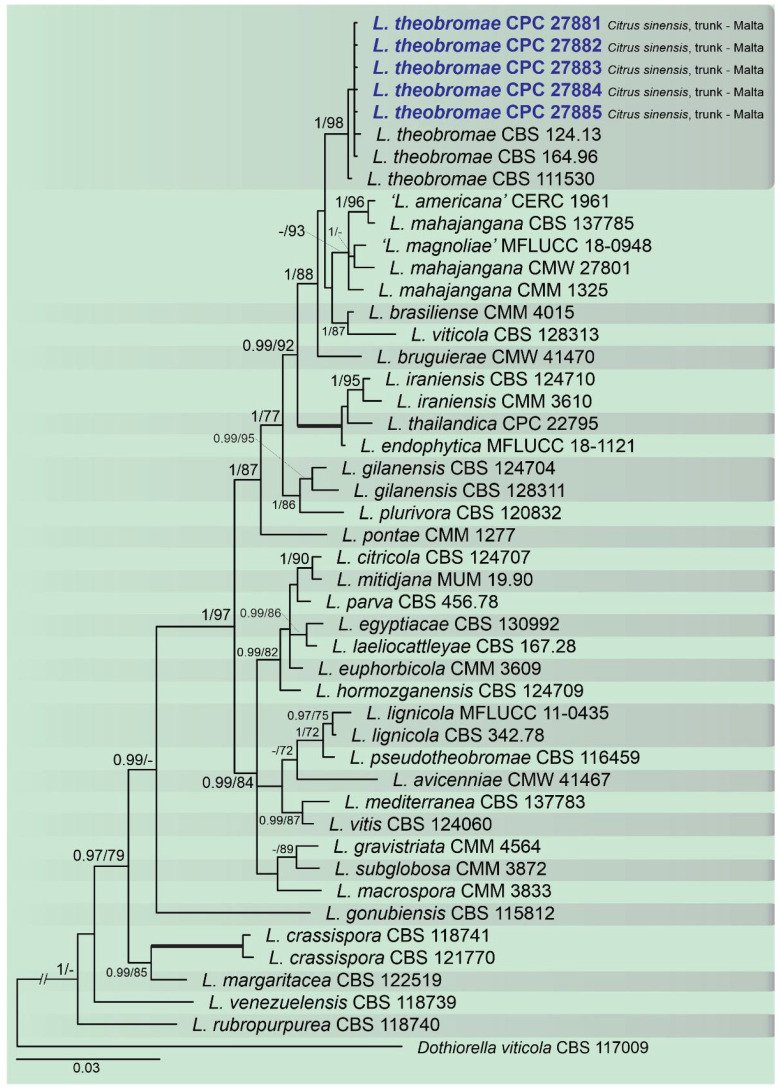
Bayesian inference analysis of *Lasiodiplodia* species using ITS rDNA, *TEF1*, and *TUB2* sequences. Isolates obtained in this study are in bold and blue. Bayesian posterior probability (BPP) and ML bootstrap (ML-BS) values equal or greater than 0.95 and 70%, respectively, are shown near nodes. Thickened branches represent clades with ML-BS = 100% and a BPP = 1.0. The tree was rooted to *Do. viticola* (CBS 117009).

**Figure 5:**
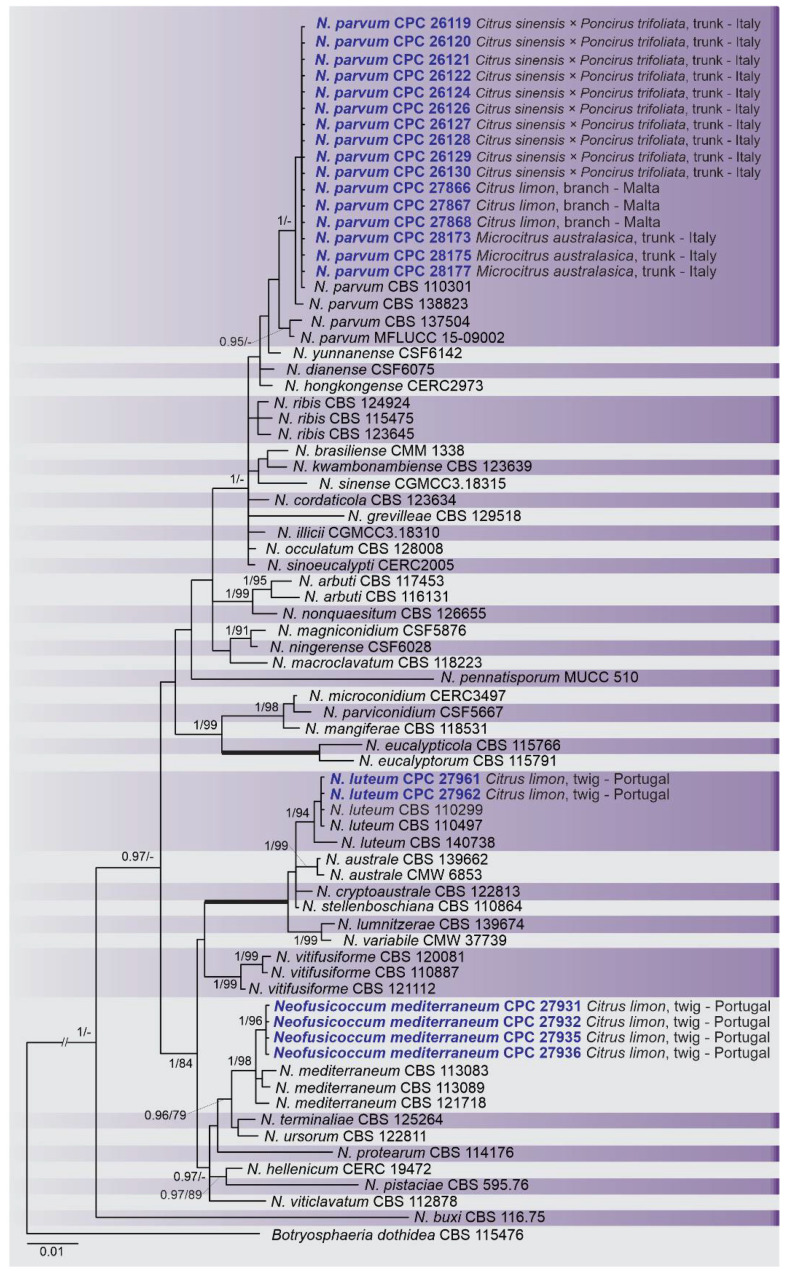
Bayesian inference analysis of species *Neofusicoccum* using ITS rDNA, *TEF1*, and *TUB2* sequences. Isolates obtained in this study are in bold and blue. Bayesian posterior probability (BPP) and ML bootstrap (ML-BS) values equal or greater than 0.95 and 70%, respectively, are shown near nodes. Thickened branches represent clades with ML-BS = 100% and a BPP = 1.0. The tree was rooted to *B. dothidea* (CBS 115476).

A combined multi-marker (ITS, *TEF1*, and *TUB2*) phylogenetic tree was inferred for each genus (*Diplodia*, *Dothiorella*, *Lasiodiplodia*, and *Neofusicoccum*) obtained in this study (Figure 2, Figure 3, Figure 4 and Figure 5). The best nucleotide models for the Bayesian Inference analysis of each dataset were as follows: SYM (symmetrical model) + I (proportion of invariable sites) + G (gamma distribution) (*Diplodia*, *Dothiorella*, *Lasiodiplodia*, and *Neofusicoccum*) for ITS; GTR (generalized time-reversible model) + G (*Diplodia*, *Dothiorella* and *Neofusicoccum*) and HKY (Hasegawa–Kishino–Yano) + I + G (*Lasiodiplodia*) for *TEF1* and GTR + G (*Diplodia*, *Lasiodiplodia* and *Neofusicoccum*) and GTR + I + G (*Dothiorella*) for *TUB2*. The *Diplodia* phylogenetic analysis revealed the isolates as belonging to *Di. pseudoseriata* (15 isolates, BPP = 1 and ML-BS = 100), *Di. seriata* (9 isolates, BPP = 1 and ML-BS = 95), *Di. olivarum* (2 isolates, Bayesian posterior probabilities (BPP) = 1 and maximum likelihood bootstrapped (ML-BS) = 99), and *Di. mutila* (1 isolate, BPP = 0.99 and ML-BS = 87) (Figure 2). The *Dothiorella* phylogeny (Figure 3) grouped the isolates together within *Do. viticola* (9 isolates, BPP = 1 and ML-BS = 99). The *Lasiodiplodia* phylogenetic analysis placed five isolates as *L. theobromae* (BPP = 1 and ML-BS = 98) (Figure 4). The *Neofusicoccum* phylogeny (Figure 5) grouped sequences from our isolates as belonging to *N. luteum* (2 isolates, BPP = 1 and ML-BS = 94), *N. parvum* (16 isolates) and *N. mediterraneum* (4 isolates, BPP = 1 and ML-BS = 98).

### 2.3. Occurrence of Botryosphaeriaceae among Countries and Citrus Species

Among countries, *Do. viticola* was found in Greece, Italy, Portugal, and Spain; *N. parvum* in Italy and Malta, and *Di. pseudoseriata* in Portugal and Spain. In addition, *Di. mutila* and *Di. seriata* were exclusively isolated in Greece and Spain, respectively; *L. theobromae* and *Di. olivarum* were only found in Malta, and *N. luteum* and *N. mediterraneum* were exclusively found in Portugal. Based on the citrus species, *N. parvum* (25.4%) and *Di. pseudoseriata* (23.8%) were the most frequently detected Botryosphaeriaceae spp. on *C. sinensis* × *P. trifoliata*, *C. limon*, *C. reticulata*, *C. sinensis*, and/or *M. australasica*; *Di. seriata* (on *C. reticulata* and *C. sinensis*); and *Do. viticola* (on *C. aurantium* and *C. sinensis*) had an equal percentage of frequency (14.3%); *Di. mutila* (exclusively found on *C. sinensis*), *N. luteum* and *N. mediterraneum* (only found on *C. limon*) and *Di. olivarum* and *L. theobromae* (exclusively found on *C. sinensis*) had low frequency values varying from 1.6% to 7.9%.

### 2.4. Pathogenicity Tests

**Figure 6:**
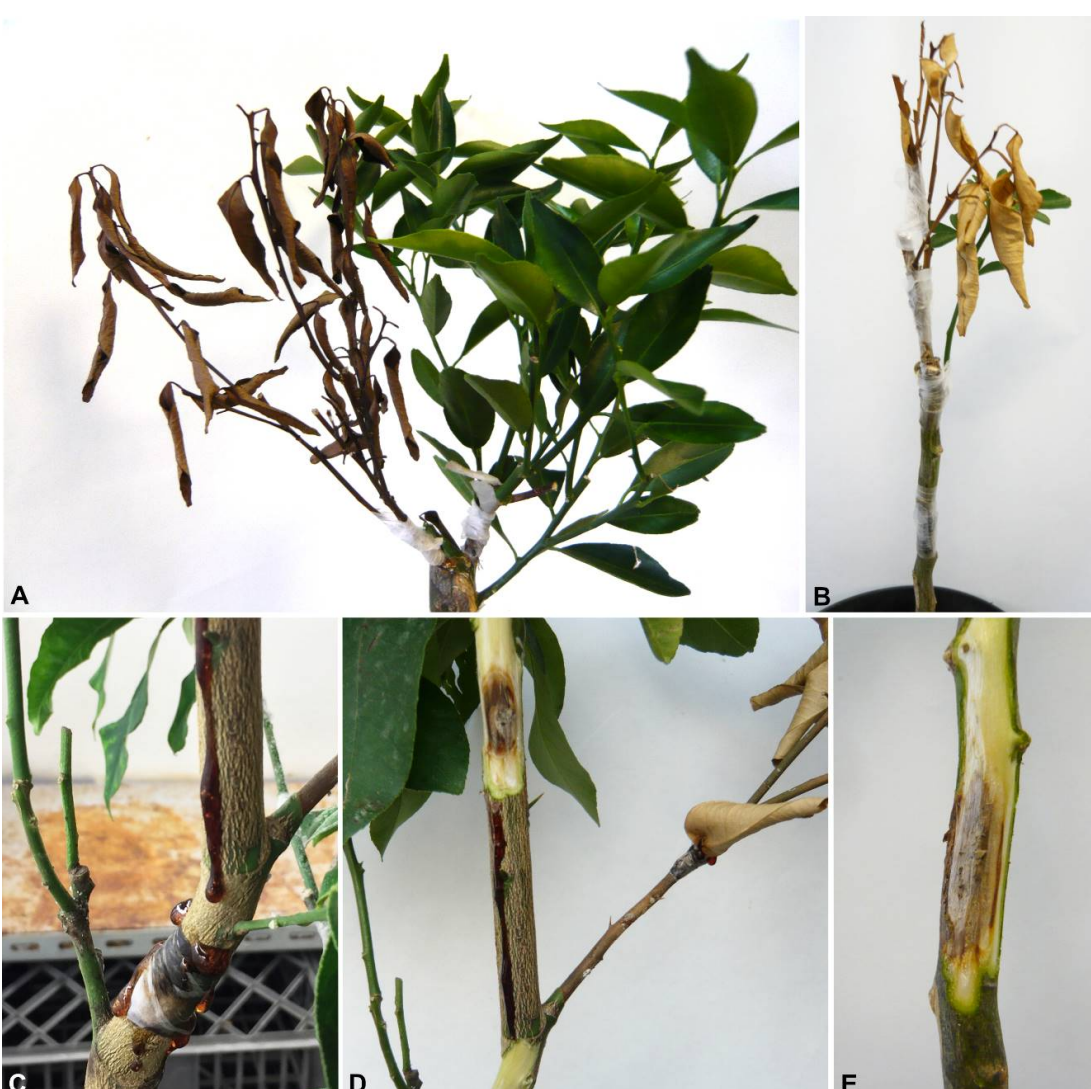
Pathogenicity tests of selected Botryosphaeriacae isolates on citrus plants 60 d after inoculation. (**A**,**B**) Shoot blight of *C. reticulata* and *C. sinensis* plants inoculated with *N*. *mediterraneum*. (**C**) Internal lesion with abundant gummosis of *C. sinensis* plant caused by *N*. *parvum*. (**D**,**E**) Internal discoloration of *C. sinensis* and *C. reticulata* twigs inoculated with *L*. *theobromae*.

**Figure 7:**
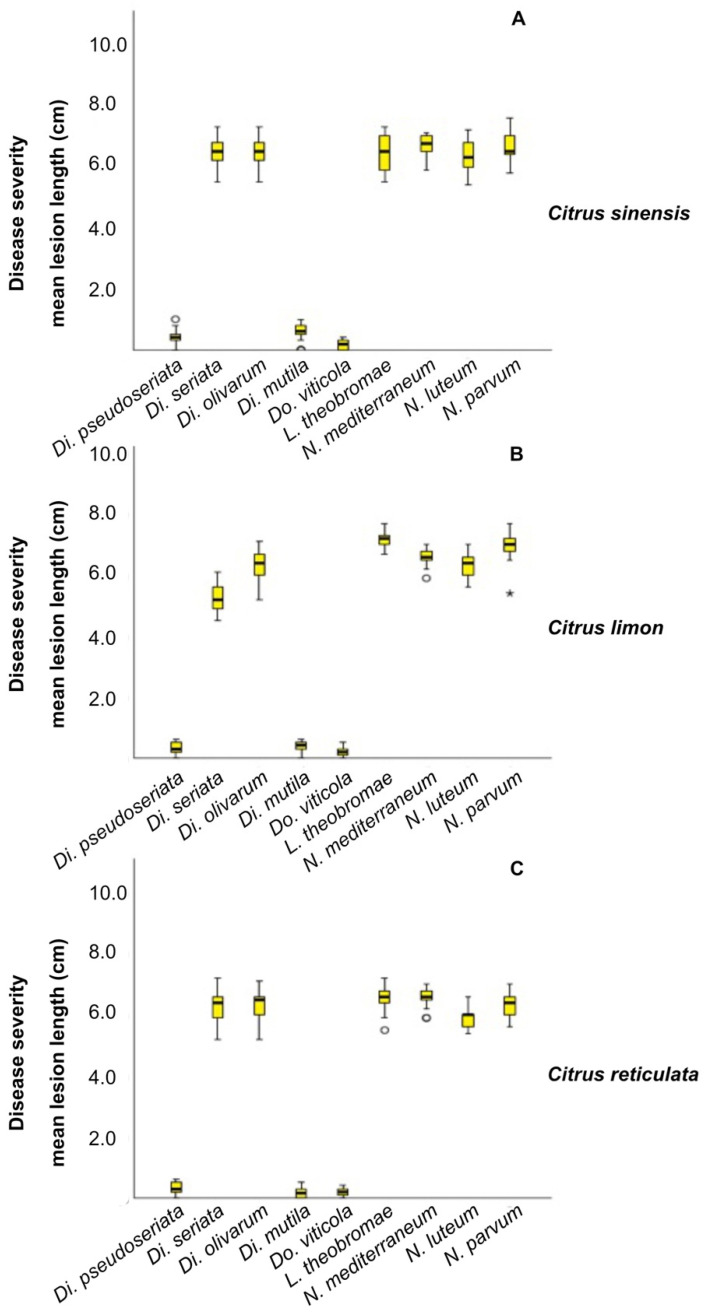
Box plot showing the results of the pathogenicity tests. Boxes represent the interquartile range, while the horizontal line within each box indicates the average value. The Kruskal–Wallis test was carried out to compare the mean lesion lengths (cm) from inoculation with nine Botryosphaeriaceae representative isolates on *C. sinensis* (**A**), *C. limon* (**B**) and *C. reticulata* (**C**). *p* < 0.05 was taken to indicate a significant difference. °: Outliers. *: Extreme values.

All isolates caused lesions on wood of inoculated plants 60 d after inoculation (Figure 6) and the fungi were successfully re-isolated. No lesions were observed on control plants. The frequency of re-isolation was between 90% and 95%. The identities of the respective inoculated and re-isolated species were confirmed using culture and molecular analysis as described above, fulfilling Koch’s postulates. Lesions and internal discolouration were observed in correspondence to the inoculation points (Figure 7). The inoculated species that showed high aggressiveness on *C*. *sinensis*, *C*. *limon*, and *C*. *reticulata* were *Di*. *seriata*, *Di*. *olivarum*, *L*. *theobromae*, *N*. *mediterraneum*, *N*. *luteum*, and *N*. *parvum* (with mean lesion length (MLL) ranged from 5.25 to 6.96 cm). Weak symptoms were caused by *Di*. *pseudoseriata*, *Di*. *mutila*, and *Do*. *viticola* on the same species (with MLL ranged from 0.17 to 0.58 cm).

**Figure 8:**
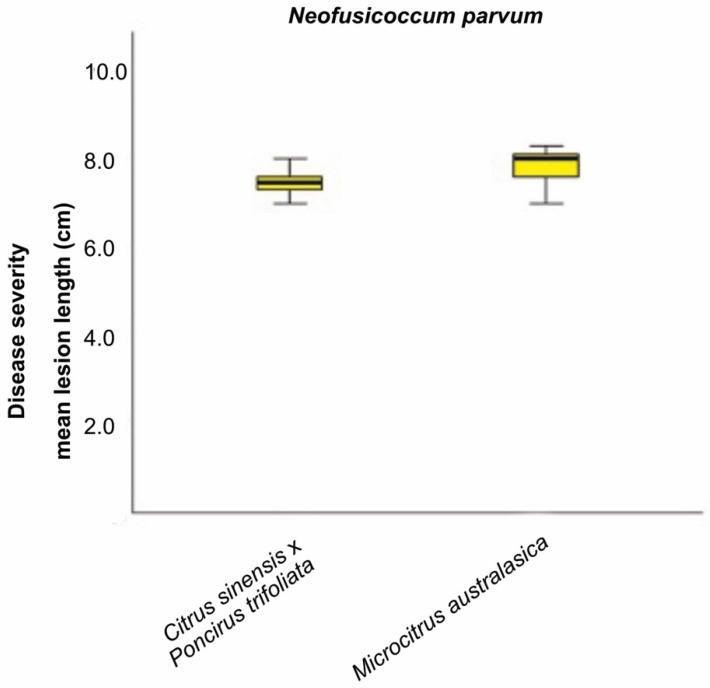
The Kruskal–Wallis test was carried out to compare the mean lesion lengths (cm) from inoculation with one *N. parvum* representative isolate on *C. sinensis* × *P. trifoliata* and *M. australasica.* Significant difference was accepted for *p* < 0.05.

For each tested host species, the pairwise comparison, obtained from the Kruskal–Wallis test, showed significant differences (*p* < 0.05) between the species *Di*. *seriata*, *Di*. *olivarum*, *L*. *theobromae*, *N*. *mediterraneum*, *N*. *luteum*, and *N*. *parvum* and the remaining pathogens *Di*. *pseudoseriata*, *Di*. *mutila* and *Do*. *viticola* (Supplementary Tables S1–S3). No significant differences were observed within the group composed by *Di*. *seriata*, *Di*. *olivarum*, *L*. *theobromae*, *N*. *mediterraneum*, *N*. *luteum*, and *N*. *parvum*. Moreover, *N*. *parvum* revealed to be highly aggressive on *M. australasica* and *C. sinensis* x *P. trifoliata* with similar level of aggressiveness (Figure 8). The tested strain developed a MLL = 7.83 cm on *M. australasica* and a MLL = 7.45 cm on *C. sinensis* × *P. trifoliata*.

## 3. Discussion

Several Botryosphaeriaceae spp. have been detected in association with citrus cankers worldwide. *Diplodia seriata*, *Di. mutila*, *Do. iberica*, *Do. viticola*, *L. parva*, *N. australe*, *N. luteum*, *N. mediterraneum*, *N. parvum*, and *Ne. dimidiatum* have been recovered from necrotic tissues of branch canker and rootstock citrus samples in California [13,31,36]. Recently, *Di. citricarpa* was described for a fungus on twigs of *Citrus* sp. in Iran [16] and *L. mitidjana* was introduced for a fungus causing branch canker and dieback of *C. sinensis* in Algeria [33]. Botryosphaeriaceae spp. causing disease on citrus are known in European countries, where *N. parvum* and *Ne. dimidiatum* were reported on *C. reticulata* in Greece and on *C. sinensis* in Italy, respectively [32,37]. 

This study represents the first large survey aimed at studying the occurrence, genetic diversity, and pathogenicity of Botryosphaeriaceae species associated with symptomatic citrus species of citrus-producing areas in Greece, Italy, Portugal, Malta, and Spain [10,38]. Results obtained during our study have added new information about the pathogenicity of Botryosphaeriaceae spp. in citrus-producing areas of these European countries. Symptomatic plants were observed during fieldwork in all the citrus orchards and regions investigated and all isolates used in the pathogenicity test caused lesions on wood of inoculated citrus plants. Phylogenetic multi-marker analyses recognized botryosphaeriaceous isolates in four *Diplodia* species, with *Di. pseudoseriata* (15 isolates) being the most common; followed by three *Neofusicoccum* species, with *N. parvum* (16 isolates) as dominant species, *Do. viticola* (9 isolates), and *L. theobromae* (5 isolates). All species found in this study, except *Di. pseudoseriata* and *Di. olivarum*, which are reported for the first time on *Citrus* spp., have been found in citrus-producing areas of California (USA) [13,31,36]. 

*Diplodia* and *Neofusicoccum* species were dominant in this study. Different species of *Neofusicoccum* and *Diplodia* were the most frequently detected pathogens causing gummosis on citrus in California [36] and *Di. citricarpa* was a new species isolated from *Citrus* sp. in Iran [16]. Species of *Diplodia*, *Dothiorella*, *Lasiodiplodia*, and *Neofusicoccum* detected in our study are widely reported as pathogens of other host plants in Algeria and Tunisia [39,40], Australia [41], Brazil [42], China [43,44], Chile [45], Italy, Portugal [39,46,47,48], South Africa [49], and the USA [13,31,36]. The results obtained in our study provide valuable information related to the richness, occurrence, and pathogenicity of Botryosphaeriaceae species in association with citrus species. This study is also the first major survey for Botryosphaeriaceae species associated with symptomatic citrus species in citrus-producing areas of five European countries, providing essential information for future monitoring. Moreover, while previous reports of canker diseases of citrus were based exclusively on morphological observations, the current study aimed to investigate the fungi affecting the major citrus production areas in Europe by large-scale sampling, morphology, and DNA phylogeny. The information achieved with this study about Botryosphaeriaceae population and citrus canker etiology provide fundamental knowledge to start further studies aimed to improve the disease management.

## 4. Materials and Methods

### 4.1. Field Sampling and Fungal Isolation

During 2015 and 2016 more than 90 sites in the most important citrus-producing areas of Europe were investigated. The surveys were conducted in Andalusia, Valencia, and the Balearic Islands (Spain); Apulia, Calabria, Sicily, and Aeolian Islands (Italy); Algarve (Portugal); Arta, Crete, Missolonghi, and Nafplio (Greece); Malta and Gozo (Malta) [10,38]. Twig, branch and trunk portions showing cankers and dieback were collected. Investigated species of *Citrus* and allied genera of the Rutaceae family such as *Microcitrus* included: *C. limon*, *C. reticulata*, *C. sinensis*, *M. australasica*, and *C. sinensis* × *P. trifoliata.*

Wood fragments (5 × 5 mm) were collected from the margin between necrotic and healthy tissues. Then, each fragment was disinfected by immersion in 70% ethanol for 5 s, 4% sodium hypochlorite for 90 s, sterilised distilled water for 60 s and then dried on sterile filter paper. The fragments were placed into Petri dishes containing malt extract agar (MEA) [50] supplemented with penicillin (100 μg/mL) and streptomycin (100 μg/mL) (MEA-PS) and incubated at 25 °C until characteristic Botryosphaeriaceae colonies were observed. A second procedure was used with plant material incubated in moist chambers at 20 ± 3 °C for up to 10 d and inspected daily for fungal sporulation. The conidia obtained through both procedures were collected and crushed in a drop of sterile water and then spread over the surface of MEA-PS plates. After 24 h, germinating spores were individually transferred onto MEA plates. The isolates used in this study are maintained in the working collection of Pedro Crous (CPC), housed at the Westerdijk Fungal Biodiversity Institute (CBS), Utrecht, The Netherlands.

The occurrence of botryosphaeriaceous fungi among countries and citrus species was evaluated as the number of isolates from each fungal species against the total number of isolates and expressed as a percentage.

### 4.2. DNA Extraction, Polymerase Chain Reaction (PCR) Amplification and Sequencing

Colonies grown on MEA for 7 days were used to perform total DNA extraction using the Wizard^®®^ Genomic DNA Purification Kit (Promega, Madison, WI, USA) standard protocol. The primer pair ITS4/ITS5 [51] was used to amplify the ITS. The primer sets EF1-728F/EF2 [52,53] and Bt2a/Bt2b [54] were used to amplify partial fragments of the *TEF1* and *TUB2* genes, respectively. Amplification by PCR was conducted as described by Yang et al. [16]. The PCR products were sequenced in both directions using the BigDye^®®^ Terminator v. 3.1 Cycle Sequencing Kit (Applied Biosystems Life Technologies, Carlsbad, CA, USA), after which amplicons were purified through Sephadex G-50 Fine columns (GE Healthcare, Freiburg, Germany) in MultiScreen HV plates (Millipore, Billerica, MA, USA). Purified sequence reactions were analyzed on an Applied Biosystems 3730xl DNA Analyzer (Life Technologies, Carlsbad, CA, USA). The DNA sequences generated were analyzed and consensus sequences were computed using SeqMan Pro (DNASTAR, Madison, WI, USA). Sequences obtained in this study were deposited in GenBank https://academic.oup.com/nar/article/49/D1/D92/5983623 (accessed on 30 January 2021) (Table 2).

### 4.3. Phylogenetic Analyses

The phylogenetic analyses included DNA sequences generated in this study along with DNA sequences retrieved from GenBank (Table 2) and represent 124 Botryosphaeriaceae species (*Diplodia* = 23; *Dothiorella* = 31; *Lasiodiplodia* = 31; *Neofusicoccum* = 39) following recent studies [16,23,25]. Alignments were first made using MAFFT v. 7 [55] and manually checked and edited using MEGA v.7 [56]. Maximum Likelihood (ML) and Bayesian Inference (BI) analyses were conducted using RAxML-HPC BlackBox v.8.2.8 [57] and MrBayes v.3.2.7a on XSEDE, respectively, at the CIPRES Science Gateway. The best nucleotide models for the BI analysis were calculated using MrModelTest v.2.3 [58] while GTR + I + G was used for ML analysis. Clade stability of the ML phylogeny was assessed with 1000 bootstrap replicates. The BI analysis lasted for one million generations, a burning value of 25% and chains were sampled every 1000 generations. Values of ML bootstrap (ML-BS) and BI posterior probability (BPP) equal or greater than 70% and 0.95, respectively, were considered significant. Individual gene phylogenies were visually inspected and compared for topological incongruences before combining into a multi-marker sequence alignment. The combined alignments used to perform the phylogenetic inferences were deposited in TreeBASE (study ID S27709).

### 4.4. Pathogenicity Tests

Pathogenicity tests with nine Botryosphaeriaceae species isolated from the European citrus samples were performed to satisfy Koch’s postulates. 

One isolate of *Di. pseudoseriata* (CPC 28084), *Di. seriata* (CPC 28091), *Di. olivarum* (CPC 27855), *Di. mutila* (CPC 26977), *Do. viticola* (CPC 27125), *L. theobromae* (CPC 27881), *N. mediterraneum* (CPC 27931), *N. luteum* (CPC 27961), and *N. parvum* (CPC 28175) were respectively inoculated onto potted 2-y-old healthy plants of lemon (*C. limon*), mandarin (*C. reticulata*) and sweet orange (*C. sinensis*). The strain of *N. parvum* was also inoculated onto potted 2-y-old healthy plants of Australasian lime (*M. australasica*) and Carrizo citrange (*C. sinensis × P. trifoliata*).

Three plants for each isolate were inoculated, each having five wounds on twigs made using a sterile blade. Mycelial plugs (5 mm diam.), taken from the margin of actively growing colonies on MEA, were placed on the wound sites on each plant. An equivalent number of plants and inoculation sites were inoculated with sterile MEA plugs and served as controls. The inoculation sites were covered with Parafilm^®®^ (American National Can, Chicago, IL, USA). The inoculated plants were incubated with a 16 h photoperiod in a growth chamber at 100% relative humidity and 25 ± 1 °C. After 2 months external symptoms were assessed. Twigs were cut and the bark peeled off to check for any internal discolouration and the total, upward and downward lesion length was taken to evaluate the MLL. Small sections (0.5 cm) of symptomatic tissue from the edge of twig lesions were placed on MEA to re-isolate the fungal species and were identified based on *TEF1* sequencing to fulfil Koch’s postulates. The experiment was conducted twice and each trial was considered a replicate. Because no normal distribution was observed in the lesion dimension data, the Kruskal–Wallis non-parametric test (at *P* = 0.05) was performed to determine significant differences among isolates. The data analysis was conducted using SPSS software 26 (IBM Corporate).

## Figures and Tables

**Table 1:** Geographical sites investigated and sampled.

Site	Locality	GPS Coordinates
1	Algemesi (Spain)	39°11′48.8″ N, 0°28′15.0″ W
2	Alginet (Spain)	39°15′36.3″ N, 0°27′28.9″ W
3	Alhaurin El Grande (Spain)	36°38′43.4″ N, 4°40′37.5″ W
4	Alzira (Spain)	39°09′25.1″ N, 0°29′26.6″ W
5	Castellò (Spain)	39°54′14.1″ N, 0°05′10.3″ W
6	Estellencs (Spain)	39°39′12.6″ N, 2°28′54.8″ E
7	Faro (Portugal)	37°03′45.5″ N, 7°55′02.8″ W
8	Gozo (Malta)	36°02′15.1″ N, 14°15′36.4″ E
9	Gozo (Malta)	36°03′18.5″ N, 14°15′35.7″ E
10	Malaga (Spain)	36°45′42.3″ N, 4°25′37.4″ W
11	Mascali (Italy)	37°46′05.7″ N, 15°11′40.7″ E
12	Massafra (Italy)	40°32′41.1″ N, 17°08′38.8″ E
13	Mastro (Greece)	38°25′49.0″ N, 21°16′49.9″ E
14	Mesquita (Portugal)	37°12′16.3″ N, 8°17′52.1″ W
15	Moncada (Spain)	39°35′18.8″ N, 0°23′40.5″ W
16	Nafplio (Greece)	37°34′56.3″ N, 22°41′48.5″ E
17	Rocca Imperiale (Italy)	40°06′30.2″ N, 16°37′04.6″ E
18	Scordia (Italy)	37°16′53.5″ N, 14°52′08.9″ E
19	Silves (Portugal)	37°09′50.7″ N, 8°23′21.7″ W

**Table 2:** GenBank accession numbers of sequences of *Diplodia*, *Dothiorella*, *Lasiodiplodia*, and *Neofusicoccum* species used in the phylogenetic analyses. Isolates and sequences obtained in this study are given in bold.

Species	Strains ^1^	Host/Substrate	Country	GenBank Numbers ^2^		
				ITS	*TEF1*	*TUB2*
*Botryosphaeria dothidea*	CBS 115476 = CMW 8000, ex-epitype	*Prunus* sp.	Switzerland	AY236949	AY236898	AY236927
*Diplodia africana*	CBS 120835 = CPC 5908, ex-type	*Prunus persica*, stem canker	South Africa	EF445343	EF445382	KF766129
*Di. agrifolia*	CBS 132777 = UCR732, ex-type	*Quercus agrifolia*, cankered branch	USA: California	JN693507	JQ517317	JQ411459
*Di. allocellula*	CBS 130408 = CMW 36468, ex-type	*Acacia karroo*, healthy branches	South Africa	JQ239397	JQ239384	JQ239378
*Di. bulgarica*	CBS 124254 = CAP332, ex-type	*Malus sylvestris*	Bulgaria	GQ923853	GQ923821	–
*Di. citricarpa*	CBS 124715 = CJA 131 = IRAN 1578C, ex-type	*Citrus* sp., twigs	Iran	KF890207	KF890189	KX464784
*Di. corticola*	CBS 112549 = CAP 134, ex-type	*Quercus suber*	Portugal	AY259100	AY573227	DQ458853
*Di. crataegicola*	MFLU 15-13112, ex-type	*Crataegus* sp.	Italy	KT290244	KT290248	KT290246
*Di. cupressi*	CBS 168.87, ex-type	*Cupressus sempervirens*, canker	Israel	DQ458893	DQ458878	DQ458861
*Di. eriobotryicola*	CBS 140851 = BN-21, ex-type	*Eriobotrya japonica*	Spain	KT240355	KT240193	MG015806
*Di. estuarina*	CMW 41231	*Avicennia marina*	South Africa	KP860831	KP860676	KP860754
*Di. fraxini*	CBS 136010 = CAD001, ex-type	*Fraxinus angustifolia*	Portugal	KF307700	KF318747	MG015807
*Di. gallae*	CBS 211.25	*Quercus* sp., fruit	–	KX464090	KX464564	KX464795
*Di. gallae*	CBS 212.25	*Quercus* sp., gall	–	KX464091	KX464565	KX464796
*Di. gallae*	CBS 213.25	*Quercus* sp., gall	–	KX464092	KX464566	KX464797
*Di. malorum*	CBS 124130 = CAP271, ex-type	*Malus sylvestris*	Portugal	GQ923865	GQ923833	–
***Di. mutila***	**CPC 26977**	***Citrus sinensis*** **, twig**	**Greece**	**MW413831**	**MW419149**	**MW419212**
*Di. mutila*	CBS 112553 = CAP 062	*Vitis vinifera*	Portugal	AY259093	AY573219	DQ458850
*Di. mutila*	CBS 121862 = PD 03708098, ex-type of *Di. pyri*	*Pyrus* sp.	The Netherlands	KX464093	KX464567	KX464799
*Di. neojuniperi*	CPC 22753 = B0031, ex-type	*Juniperus chinensis*	Thailand	KM006431	KM006462	–
***Di. olivarum***	**CPC 27855**	***Citrus sinensis*** **,** **branch**	**Malta**	**MW413832**	**MW419150**	**MW419213**
***Di. olivarum***	**CPC 27856**	***Citrus sinensis*** **,** **branch**	**Malta**	**MW413833**	**MW419151**	**MW419214**
*Di. olivarum*	CBS 121886	*Olea europaea*	Italy	EU392301	EU392278	–
*Di. olivarum*	CBS 121887 = CAP 254, ex-type	*Olea europaea*, rotting drupes	Italy	EU392302	EU392279	HQ660079
*Di. pseudoseriata*	CBS 124906, ex-type	*Blepharocalyx salicifolius*	Uruguay	EU080927	EU863181	MG015820
***Di. pseudoseriata***	**CPC 27963**	***Citrus sinensis*** **, twig**	**Portugal**	**MW413834**	**MW419152**	**MW419215**
***Di. pseudoseriata***	**CPC 27964**	***Citrus sinensis*** **, twig**	**Portugal**	**MW413835**	**MW419153**	**MW419216**
***Di. pseudoseriata***	**CPC 27965**	***Citrus sinensis*** **, twig**	**Portugal**	**MW413836**	**MW419154**	**MW419217**
***Di. pseudoseriata***	**CPC 27966**	***Citrus sinensis*** **, twig**	**Portugal**	**MW413837**	**MW419155**	**MW419218**
***Di. pseudoseriata***	**CPC 27967**	***Citrus sinensis*** **, twig**	**Portugal**	**MW413838**	**MW419156**	**MW419219**
***Di. pseudoseriata***	**CPC 28084**	***Citrus reticulata*** **, twig**	**Spain**	**MW413839**	**MW419157**	**MW419220**
***Di. pseudoseriata***	**CPC 28086**	***Citrus reticulata*** **, twig**	**Spain**	**MW413840**	**MW419158**	**MW419221**
***Di. pseudoseriata***	**CPC 28087**	***Citrus reticulata*** **, twig**	**Spain**	**MW413841**	**MW419159**	**MW419222**
***Di. pseudoseriata***	**CPC 28092**	***Citrus limon*** **, twig**	**Spain**	**MW413842**	**MW419160**	**MW419223**
***Di. pseudoseriata***	**CPC 28093**	***Citrus limon*** **, twig**	**Spain**	**MW413843**	**MW419161**	**MW419224**
***Di. pseudoseriata***	**CPC 28094**	***Citrus limon*** **, twig**	**Spain**	**MW413844**	**MW419162**	**MW419225**
***Di. pseudoseriata***	**CPC 28095**	***Citrus limon*** **, twig**	**Spain**	**MW413845**	**MW419163**	**MW419226**
***Di. pseudoseriata***	**CPC 28099**	***Citrus reticulata*** **, twig**	**Spain**	**MW413846**	**MW419164**	**MW419227**
***Di. pseudoseriata***	**CPC 28100**	***Citrus reticulata*** **, twig**	**Spain**	**MW413847**	**MW419165**	**MW419228**
***Di. pseudoseriata***	**CPC 28102**	***Citrus reticulata*** **, twig**	**Spain**	**MW413848**	**MW419166**	**MW419229**
*Di. pseudoseriata*	BL132	*Fraxinus angustifolia*	Italy	KF307720	KF318767	MG015810
*Di. pseudoseriata*	CBS 140350, ex-type of *Di. insularis*	*Pistacia lentiscus*	Italy	KX833072	KX833073	MG015809
*Di. pseudoseriata*	CBS 124931 = CMW22627, ex-type of *Di. alatafructa*	*Pterocarpus angolensis*, bark wound	South Africa	FJ888460	FJ888444	MG015799
*Di. quercivora*	CBS 133852 = BL8, ex-type	*Quercus canariensis*	Tunisia	JX894205	JX894229	MG015821
*Di. rosulata*	CBS 116470, ex-type	*Prunus africana*	Ethiopia	EU430265	EU430267	EU673132
*Di. sapinea*	CBS 393.84, ex-epitype	*Pinus nigra*, cones	Netherlands	DQ458895	DQ458880	DQ458863
*Di. sapinea*	CBS 124462 = CAP273, ex-type of *Di. intermedia*	*Malus sylvestris*	Portugal	GQ923858	GQ923826	–
*Di. sapinea*	CBS 141915 = NB7, ex-type of *Di. rosacearum*	*Eriobotrya japonica*	Italy	KT956270	KU378605	MG015823
*Di. scrobiculata*	CBS 118110 = CMW 189 = BOT 1195, ex-type	*Pinus banksiana*	USA: Wisconsin	AY253292	AY624253	AY624258
*Di. seriata*	CBS 112555 = HAP 052 = CAP 063, ex-epitype	*Vitis vinifera*, dead stems	Portugal	AY259094	AY573220	DQ458856
***Di. seriata***	**CPC 28088**	***Citrus reticulata*** **,** **twig**	**Spain**	**MW413849**	**MW419167**	**MW419230**
***Di. seriata***	**CPC 28089**	***Citrus reticulata*** **,** **twig**	**Spain**	**MW413850**	**MW419168**	**MW419231**
***Di. seriata***	**CPC 28090**	***Citrus reticulata*** **,** **twig**	**Spain**	**MW413851**	**MW419169**	**MW419232**
***Di. seriata***	**CPC 28091**	***Citrus reticulata*** **,** **twig**	**Spain**	**MW413852**	**MW419170**	**MW419233**
***Di. seriata***	**CPC 28096**	***Citrus sinensis*** **,** **twig**	**Spain**	**MW413853**	**MW419171**	**MW419234**
***Di. seriata***	**CPC 28097**	***Citrus sinensis*** **,** **twig**	**Spain**	**MW413854**	**MW419172**	**MW419235**
***Di. seriata***	**CPC 28098**	***Citrus sinensis*** **,** **twig**	**Spain**	**MW413855**	**MW419173**	**MW419236**
***Di. seriata***	**CPC 28101**	***Citrus reticulata*** **,** **twig**	**Spain**	**MW413856**	**MW419174**	**MW419237**
***Di. seriata***	**CPC 28103**	***Citrus reticulata*** **,** **twig**	**Spain**	**MW413857**	**MW419175**	**MW419238**
*Di. seriata*	CBS 119049	*Vitis* sp.	Italy	DQ458889	DQ458874	DQ458857
*Di. subglobosa*	CBS 124133 = JL453, ex-type	*Lonicera nigra*	Spain	GQ923856	GQ923824	–
*Di. tsugae*	CBS 418.64 = IMI 197143, ex-isotype	*Tsuga heterophylla*	Canada	DQ458888	DQ458873	DQ458855
*Dothiorella alpina*	CGMCC 3-18001, ex-type	*Platycladus orientalis*	China	KX499645	KX499651	–
*Do. americana*	CBS 128309, ex-type	Wedge-shape canker of grapevine cv. Vignoles (complex hybrid of North America *Vitis* species and *Vitis vinifera*)	USA: Missouri	HQ288218	HQ288262	HQ288297
*Do. brevicollis*	CBS 130411 = CMW 36463, ex-type	*Acacia karroo*, healthy branches	South Africa	JQ239403	JQ239390	JQ239371
*Do. capri-amissi*	CBS 121763 = CMW 25403 = CAMS 1158, ex-paratype	*Acacia erioloba*	South Africa	EU101323	EU101368	KX464850
*Do. capri-amissi*	CBS 121878 = CMW 25404 = CAMS 1159, ex-type	*Acacia erioloba*	South Africa	EU101324	EU101369	KX464851
*Do. casuarinae*	CBS 120688 = CMW 4855, ex-type	*Casuarina* sp.	Australia: Australian Capital Territory	DQ846773	DQ875331	DQ875340
*Do. casuarinae*	CBS 120689 = CMW 4856, ex-paratype	*Casuarina* sp.	Australia: Australian Capital Territory	DQ846772	DQ875332	DQ875339
*Do. casuarinae*	CBS 120690 = CMW 4857, ex-paratype	*Casuarina* sp.	Australia: Australian Capital Territory	DQ846774	DQ875333	DQ875341
*Do. citricola*	CBS 124728 = ICMP 16827	*Citrus sinensis*	New Zealand	EU673322	EU673289	KX464852
*Do. citricola*	CBS 124729 = ICMP 16828, ex-type	*Citrus sinensis*, twigs	New Zealand	EU673323	EU673290	KX464853
*Do. dulcispinae*	CBS 121764 = CMW 25406 = CAMS 1159, ex-paratype of *Dothiorella oblonga*	*Acacia mellifera*	Namibia	EU101299	EU101344	KX464854
*Do. dulcispinae*	CBS 130413 = CMW 36460, ex-type	*Acacia karroo*, dieback branches	South Africa	JQ239400	JQ239387	JQ239373
*Do. iberica*	CBS 113188 = DA-1	*Quercus suber*, branch canker	Spain	AY573198	EU673278	EU673097
*Do. iberica*	CBS 113189 = DE-14	*Quercus ilex*, branch canker	Spain	AY573199	AY573230	KX464855
*Do. iberica*	CBS 115041 = CAP 145, ex-type	*Quercus ilex*, dead twigs	Spain	AY573202	AY573222	EU673096
*Do. iranica*	CBS 124722 = CJA 153 = IRAN 1587C	*Olea* sp., twigs	Iran	KC898231	KC898214	KX464856
*Do. longicollis*	CBS 122066 = CMW 26164	*Terminalia* sp.	Australia: Western Australia	EU144052	EU144067	KX464857
*Do. longicollis*	CBS 122067 = CMW 26165	*Lysiphyllum cunninghamii*	Australia: Western Australia	EU144053	EU144068	KX464858
*Do. longicollis*	CBS 122068 = CMW 26166, ex-type	*Lysiphyllum cunninghamii*	Australia: Western Australia	EU144054	EU144069	KF766130
*Do. mangifericola*	CBS 124727 = IRAN 1584C = CJA 261, ex-type	*Mangifera indica*, twigs	Iran	KC898221	KX464614	–
*Do. moneti*	WAC 13154 = MUCC 505, ex-type	*Acacia rostellifera*, healthy stem	Australia: Western Australia	EF591920	EF591971	EF591954
*Do. neclivorem*	DAR 80992, ex-type	*Vitis vinifera* cv. Chardonnay, berries	Australia: New South Wales	KJ573643	KJ573640	KJ577551
*Do. oblonga*	CBS 121765 = CMW 25407 = CAMS 1162, ex-type	*Acacia mellifera*	South Africa	EU101300	EU101345	KX464862
*Do. oblonga*	CBS 121766 = CMW 25408 = CAMS 1163, ex-paratype	*Acacia mellifera*	South Africa	EU101301	EU101346	KX464863
*Do. omnivora*	CBS 124717 = CJA 214 = IRAN 1570C	*Juglans regia*, twigs	Iran	KC898233	KC898216	KX464865
*Do. omnivora*	CBS 392.80	–	France	KX464133	KX464626	KX464897
*Do. omnivora*	CBS 124716 = CJA 241 = IRAN 1573C	*Juglans regia*, twigs	Iran	KC898232	KC898215	KX464864
*Do. omnivora*	CBS 242.51	–	Italy	EU673317	EU673284	EU673105
*Do. omnivora*	CBS 188.87	*Juglans regia*	France	EU673316	EU673283	EU673119
*Do. parva*	CBS 124720 = CJA 27 = IRAN 1579C, ex-type	*Corylus* sp., twigs	Iran	KC898234	KC898217	KX464866
*Do. parva*	CBS 124721 = CJA 35	*Corylus* sp., twigs	Iran	KX464123	KX464615	KX464867
*Do. parva*	CBS 125580	*Corylus avellana*, branches	Austria	KX464124	KX464616	KX464868
*Do. plurivora*	CBS 124724 = CJA 254 = IRAN 1557C, ex-type	*Citrus* sp., twigs	Iran	KC898225	KC898208	KX464874
*Do. pretoriensis*	CBS 130404 = CMW 36480, ex-type	*Acacia karroo*, branches with dieback	South Africa	JQ239405	JQ239392	JQ239376
*Do. prunicola*	CBS 124723 = CAP 187 = IRAN 1541C, ex-type	*Prunus dulcis*, necrotic twigs	Portugal	EU673313	EU673280	EU673100
*Do. rosulata*	CBS 121760 = CMW 25389 = CAMS 1444, ex-type	*Acacia karroo*	Namibia	KF766227	EU101335	KX464877
*Do. rosulata*	CBS 121761 = CMW 25392 = CAMS 1147, ex-paratype	*Acacia mellifera*	South Africa	EU101293	EU101338	KX464878
*Do. rosulata*	CBS 121762 = CMW 25395 = CAMS 1150	*Acacia mellifera*	South Africa	EU101319	EU101364	KX464879
*Do. rosulata*	CBS 500.72	*Medicago sativa*, stubble	South Africa	EU673318	EU673285	EU673118
*Do. santali*	WAC 13155 = MUCC 509, ex-type	*Santalum acuminatum*, healthy stem	Australia: Western Australia	EF591924	EF591975	EF591958
*Do. sarmentorum*	IMI 63581b, ex-type of *Bot. sarmentorum*	*Ulmus* sp.	UK: England	AY573212	AY573235	EU673102
*Do. sempervirentis*	IRAN 1581C = CBS 124719	*Cupressus sempervirens*	Iran	KC898237	KC898220	KX464885
*Do. sempervirentis*	IRAN 1583C = CBS 124718 = CJA 264, ex-type	*Cupressus sempervirens*, twigs	Iran	KC898236	KC898219	KX464884
*Do. striata*	CBS 124730 = ICMP 16819	*Citrus sinensis*, twigs	New Zealand	EU673320	EU673287	EU673142
*Do. striata*	CBS 124731 = ICMP 16824, ex-type	*Citrus sinensis*, twigs	New Zealand	EU673321	EU673288	EU673143
*Do. thailandica*	CBS 133991 = CPC 21557 = MFLUCC 11-0438, ex-type of *Auerswaldia dothiorella*	Dead bamboo culm	Thailand	JX646796	JX646861	JX646844
*Do. thripsita*	CBS 125445 = BRIP 51876a, ex-type	*Acacia harpophylla*, dead branches, petioles & leaves	Australia: Queensland	KJ573642	KJ573639	KJ577550
*Do. uruguayensis*	CBS 124908 = CMW 26763 = UY672, ex-type	*Hexachlamis edulis*	Uruguay	EU080923	EU863180	KX464886
*Do. vidmadera*	CBS 621.74	*Pyrus communis*, leaf	Switzerland	KX464129	KX464621	KX464887
*Do. vidmadera*	CBS 725.79	*Pyrus malus*, dead wood and buds	Switzerland	KX464130	KX464622	KX464888
*Do. vinea-gemmae*	DAR 81012, ex-type	*Vitis vinifera* cv. Chardonnay, dormant buds	Australia: New South Wales	KJ573644	KJ573641	KJ577552
*Do. viticola*	CBS 117009, ex-type	*Vitis vinifera* cv. Garnatxa negra, pruned canes	Spain	AY905554	AY905559	EU673104
*Do. viticola*	DAR 80529, ex-type of *D. westralis*	*Vitis vinifera* cv. Cabernet Sauvignon, discarded canes	Australia: Western Australia	HM009376	HM800511	HM800519
***Do. viticola***	**CPC 26174**	***Citrus sinensis*** **, twig**	**Italy**	**MW413858**	**MW419176**	**MW419239**
***Do. viticola***	**CPC 26917**	***Citrus sinensis*** **, branch**	**Greece**	**MW413859**	**MW419177**	**MW419240**
***Do. viticola***	**CPC 27081**	***Citrus sinensis*** **, twig**	**Italy**	**MW413860**	**MW419178**	**MW419241**
***Do. viticola***	**CPC 27106**	***Citrus aurantium*** **, twig**	**Spain**	**MW413861**	**MW419179**	**MW419242**
***Do. viticola***	**CPC 27123**	***Citrus sinensis*** **, branch**	**Italy**	**MW413862**	**MW419180**	**MW419243**
***Do. viticola***	**CPC 27125**	***Citrus sinensis*** **, branch**	**Italy**	**MW413863**	**MW419181**	**MW419244**
***Do. viticola***	**CPC 27703**	***Citrus sinensis*** **, branch**	**Spain**	**MW413864**	**MW419182**	**MW419245**
***Do. viticola***	**CPC 27707**	***Citrus sinensis*** **, branch**	**Greece**	**MW413865**	**MW419183**	**MW419246**
***Do. viticola***	**CPC 27968**	***Citrus sinensis*** **, twig**	**Portugal**	**MW413866**	**MW419184**	**MW419247**
*Do. yunnana*	CGMCC 3-17999, ex-type	*Camellia* sp.	China	KX499643	KX499649	–
*Do. yunnana*	CGMCC 3-18000	*Camellia* sp.	China	KX499644	KX499650	–
*Dothiorella* sp.	CBS 121783 = CMW 25432 = CAMS 1187	*Acacia mearnsii*	South Africa	EU101333	EU101378	KX464859
*Dothiorella* sp.	CBS 121784 = CMW 25430 = CAMS 1185	*Acacia mearnsii*	South Africa	EU101331	EU101376	KX464860
*Dothiorella* sp.	CBS 121785 = CMW 25433 = CAMS 1188	*Acacia mearnsii*	South Africa	EU101334	EU101379	KX464861
*‘Lasiodiplodia americana’*	CERC 1961 = CFCC 50065, ex-type	*Pistacia vera* cv. Kerman, twigs	USA: Arizona	KP217059	KP217067	KP217075
*L. avicenniae*	CMW 41467 = CBS 139670, ex-type	*Avicennia marina*	South Africa	KP860835	KP860680	KP860758
*L. brasiliense*	CMM 4015 = URM 7118, ex-type	*Mangifera indica*, stems	Brazil	JX464063	JX464049	–
*L. bruguierae*	CMW 41470 = CBS 139669, ex-type	*Bruguiera gymnorrhiza*	South Africa	NR_147358	KP860678	KP860756
*L. citricola*	CBS 124707 = IRAN 1522C = CJA 72, ex-type	*Citrus* sp., twigs	Iran	GU945354	GU945340	KP872405
*L. crassispora*	CBS 118741 = WAC 12533 = CMW 14691, ex-type	*Santalum album*	Australia: Western Australia	DQ103550	EU673303	EU673133
*L. crassispora*	CBS 121770 = CMW 25414 = CAMS 1169, ex-type of *L. pyriformis*	*Acacia mellifera*	Namibia	EU101307	EU101352	–
*L. endophytica*	MFLUCC 18-1121 = KUMCC 17-0233, ex-type	*Magnolia candolii*, fresh leaves	China	MK501838	MK584572	MK550606
*L. egyptiacae*	CBS 130992 = BOT-10, ex-type	*Mangifera indica*, leaf	Egypt	JN814397	JN814424	–
*L. euphorbicola*	CMM 3609, ex-type of *L. euphorbicola*	*Jatropha curcas*, collar and root rot	Brazil	KF234543	KF226689	KF254926
*L. gilanensis*	CBS 124704 = IRAN 1523C, ex-type	*Citrus* sp., fallen twigs	Iran	GU945351	GU945342	KP872411
*L. gilanensis*	CBS 128311 = UCD 2193MO, ex-type of *L. missouriana*	Wedge-shape canker of grapevine cv. Catawba (complex hybrid of North America *Vitis* species and *Vitis vinifera*)	USA: Missouri	HQ288225	HQ288267	–
*L. gonubiensis*	CBS 115812 = CMW 14077, ex-type	*Syzygium cordatum*, twigs and leaves	South Africa	AY639595	DQ103566	DQ458860
*L. gravistriata*	CMM 4564, ex-type	*Anacardium humile*	Brazil	KT250949	KT250950	–
*L. hormozganensis*	CBS 124709 = IRAN 1500C, ex-type	*Olea* sp., twigs	Iran	GU945355	GU945343	KP872413
*L. iraniensis*	CBS 124710 = IRAN 1520C, ex-type	*Salvadora persica*, twigs	Iran	GU945346	GU945334	KP872415
*L. iraniensis*	CMM 3610, ex-type of *L. jatrophicola*	*Jatropha curcas*, collar and root rot	Brazil	KF234544	KF226690	KF254927
*L. laeliocattleyae*	CBS 167.28, ex-type of *Diplodia laeliocattleyae*	*Laeliocattleya*	Italy	KU507487	KU507454	–
*L. lignicola*	MFLUCC 11-0435 = CBS 134112, ex-type	On dead wood	Thailand	JX646797	KU887003	JX646845
*L. lignicola*	CBS 342.78, ex-type of *L. sterculiae*	*Sterculia oblonga*	Germany	KX464140	KX464634	KX464908
*L. macrospora*	CMM 3833, ex-type	*Jatropha curcas*, collar and root rot	Brazil	KF234557	KF226718	KF254941
*‘L. magnoliae’*	MFLUCC 18-0948 = KUMCC 17-0198, ex-type	*Magnolia candolii*, dead leaves	China	MK499387	MK568537	MK521587
*L. mahajangana*	CBS 124927 = CMW27801, ex-type	*Terminalia catappa*, healthy branches	Madagascar	FJ900595	FJ900641	FJ900630
*L. mahajangana*	CMM 1325, ex-type of *L. caatinguensis*	*Citrus sinensis*	Brazil	KT154760	KT008006	KT154767
*L. mahajangana*	CBS 137785 = BL104, ex-type of *L. exigua*	*Retama raetam*, branch canker	Tunisia	KJ638317	KJ638336	–
*L. margaritacea*	CBS 122519 = CMW 26162 = MOZ 11A, ex-type	*Adansonia gibbosa*	Australia: Western Australia	EU144050	EU144065	KX464903
*L. mediterranea*	CBS 137783 = BL1, ex-type	*Quercus ilex*, branch canker	Italy	KJ638312	KJ638331	–
*L. mitidjana*	MUM 19.90 = ALG111, ex-type	*Citrus sinensis*, branch canker	Algeria: Mitidja	MN104115	MN159114	–
*L. parva*	CBS 456.78, ex-type	Cassava-field soil	Colombia	EF622083	EF622063	KP872419
*L. plurivora*	CBS 120832 = CPC 5803, ex-type	*Prunus salicina*, wood canker	South Africa	EF445362	EF445395	KP872421
*L. pontae*	CMM 1277, ex-type	*Spondias purpurea*	Brazil	KT151794	KT151791	KT151797
*L. pseudotheobromae*	CBS 116459, ex-type	*Gmelina arborea*	Costa Rica	EF622077	EF622057	EU673111
*L. rubropurpurea*	CBS 118740 = WAC 12535 = CMW 14700, ex-type	*Eucalyptus grandis*, canker	Australia	DQ103553	EU673304	EU673136
*L. subglobosa*	CMM 3872, ex-type	*Jatropha curcas*, collar and root rot	Brazil	KF234558	KF226721	KF254942
*L. thailandica*	CBS 138760 = CPC 22795, ex-type	*Mangifera indica*, twigs	Thailand	KJ193637	KJ193681	–
*L. theobromae*	CBS 111530 = CPC 2095 = JT 695	*Leucospermum* sp.	USA: Hawaii	EF622074	EF622054	–
***L. theobromae***	**CPC 27881**	***Citrus sinensis*** **, trunk**	**Malta**	**MW413867**	**MW419185**	**MW419248**
***L. theobromae***	**CPC 27882**	***Citrus sinensis*** **, trunk**	**Malta**	**MW413868**	**MW419186**	**MW419249**
***L. theobromae***	**CPC 27883**	***Citrus sinensis*** **, trunk**	**Malta**	**MW413869**	**MW419187**	**MW419250**
***L. theobromae***	**CPC 27884**	***Citrus sinensis*** **, trunk**	**Malta**	**MW413870**	**MW419188**	**MW419251**
***L. theobromae***	**CPC 27885**	***Citrus sinensis*** **, trunk**	**Malta**	**MW413871**	**MW419189**	**MW419252**
*L. theobromae*	CBS 124.13	–	USA	DQ458890	DQ458875	DQ458858
*L. theobromae*	CBS 164.96, ex-neotype	Fruit along coral reef coast	Papua New Guinea	AY640255	AY640258	EU673110
*L. venezuelensis*	CBS 118739 = WAC 12539 = CMW 13511, ex-type	*Acacia mangium*, wood	Venezuela	DQ103547	EU673305	EU673129
*L. viticola*	CBS 128313 = UCD 2553AR, ex-type	Wedge-shape canker of grapevine cv. Vignoles (complex hybrid of North America *Vitis* species and *Vitis vinifera*)	USA: Arkansas	HQ288227	HQ288269	HQ288306
*L. vitis*	CBS 124060, ex-type	*Vitis vinifera*		KX464148	KX464642	KX464917
*Neofusicoccum arbuti*	CBS 117453 = CMW 13455, ex-type of *N. andinum*	*Eucalyptus* sp.	Venezuela	AY693976	AY693977	KX464923
*N. arbuti*	CBS 116131 = AR 4014, ex-type	*Arbutus menziesii*, canker	USA: Washington	AY819720	KF531792	KF531793
*N. australe*	CBS 139662 = CMW 6837, ex-type	*Acacia* sp.	Australia: Victoria	AY339262	AY339270	AY339254
*N. australe*	CMW 6853	*Sequoiadendron*	Australia	AY339263	AY339271	AY339255
*N. brasiliense*	CMM 1338, ex-type	*Mangifera indica*	Brazil	JX513630	JX513610	KC794030
*N. buxi*	CBS 116.75	*Buxus sempervirens*, leaf	France	KX464165	KX464678	–
*N. cordaticola*	CBS 123634 = CMW 13992, ex-type	*Syzygium cordatum*	South Africa	EU821898	EU821868	EU821838
*N. cryptoaustrale*	CBS 122813 = CMW 23785, ex-type	*Eucalyptus* sp., living branches and leaves	South Africa	FJ752742	FJ752713	FJ752756
*N. dianense*	CSF6075 = CGMCC3.20082, ex-type	*Eucalyptus urophylla × E. grandis* tree, twigs	China	MT028605	MT028771	MT028937
*N. eucalypticola*	CBS 115679 = CMW 6539, ex-type	*Eucalyptus grandis*	Australia	AY615141	AY615133	AY615125
*N. eucalyptorum*	CBS 115791 = CMW 10125 = BOT 24	*Eucalyptus grandis*	South Africa	AF283686	AY236891	AY236920
*N. grevilleae*	CBS 129518, ex-type	*Grevillea aurea*	Australia	JF951137	–	–
*N. hellenicum*	CERC 1947 = CFCC 50067, ex-type	*Pistacia vera* cultivar Aegina	Greece	KP217053	KP217061	KP217069
*N. hongkongense*	CERC2973 = CGMCC3.18749, ex-type	*Araucaria cunninghamii*	China	KX278052	KX278157	KX278261
*N. illicii*	CGMCC3.18310, ex-type	*Illicium verum*	China	KY350149	–	KY350155
*N. kwambonambiense*	CBS 123639 = CMW 14023, ex-type	*Syzygium cordatum*	South Africa	EU821900	EU821870	EU821840
*N. lumnitzerae*	CBS 139674 = CMW 41469, ex-type	*Lumnitzera racemosa*	South Africa	KP860881	KP860724	KP860801
***N. luteum***	**CPC 27961**	***Citrus limon*** **, twig**	**Portugal**	**MW413872**	**MW419190**	**MW419253**
***N. luteum***	**CPC 27962**	***Citrus limon*** **, twig**	**Portugal**	**MW413873**	**MW419191**	**MW419254**
*N. luteum*	CBS 110497 = CPC 4594 = CAP 037	*Vitis vinifera*, grape	Portugal	EU673311	EU673277	EU673092
*N. luteum*	CBS 110299 = LM 926 = CAP 002, ex-type	*Vitis vinifera*, cane	Portugal	AY259091	KX464688	DQ458848
*N. luteum*	CBS 140738 = CMW 41365, ex-type of *N. mangroviorum*	*Avicennia marina*	South Africa	KP860859	KP860702	KP860779
*N. macroclavatum*	CBS 118223 = CMW 15955 = WAC 12444, ex-type	*Eucalyptus globulus*, wood	Australia: Western Australia	DQ093196	DQ093217	DQ093206
*N. magniconidium*	CSF5876 = CGMCC3.20077, ex-type	*Eucalyptus urophylla × E. grandis* tree, twigs	China	MT028612	MT028778	MT028944
*N. mangiferae*	CBS 118531 = CMW 7024	*Mangifera indica*	Australia	AY615185	DQ093221	AY615173
*N. mediterraneum*	CBS 121718 = CPC 13137, ex-type	*Eucalyptus* sp., branches and leaves	Greece	GU251176	–	–
*N. mediterraneum*	CBS 113083 = CPC 5263, ex-type of *N. pistaciarum*	*Pistacia vera*	USA: California	KX464186	KX464712	KX464998
*N. mediterraneum*	CBS 113089 = CPC 5274, ex-type of *N. pistaciicola*	*Pistacia vera*	USA: California	KX464199	KX464727	KX465014
***N. mediterraneum***	**CPC 27931**	***Citrus limon*** **, twig**	**Portugal**	**MW413874**	**MW419192**	**MW419255**
***N. mediterraneum***	**CPC 27932**	***Citrus limon*** **, twig**	**Portugal**	**MW413875**	**MW419193**	**MW419256**
***N. mediterraneum***	**CPC 27935**	***Citrus limon*** **, twig**	**Portugal**	**MW413876**	**MW419194**	**MW419257**
***N. mediterraneum***	**CPC 27936**	***Citrus limon*** **, twig**	**Portugal**	**MW413877**	**MW419195**	**MW419258**
*N. microconidium*	CERC3497 = CGMCC3.18750, ex-type	*Eucalyptus urophylla × E. grandis* tree	China	KX278053	KX278158	KX278262
*N. nonquaesitum*	CBS 126655 = L3IE1 = PD484, ex-type	*Umbellularia californica*, cankered branch	USA: California	GU251163	GU251295	GU251823
*N. ningerense*	CSF6028 = CGMCC3.20078, ex-type	*Eucalyptus urophylla × E. grandis* tree, twigs	China	MT028613	MT028779	MT028945
*N. occulatum*	CBS 128008 = MUCC 227, ex-type	*Eucalyptus grandis* hybrid	Australia: Queensland	EU301030	EU339509	EU339472
*N. pandanicola*	MFLUCC 17-2270 = KUMCC 17-0184, ex-type	*Pandanus* sp.	China	MH275072	–	–
*N. parviconidium*	CSF5667 = CGMCC3.20074, ex-type	*Eucalyptus* tree, twigs	China	MT028615	MT028781	MT028947
*N. parvum*	CBS 138823 = ICMP 8003 = CMW 9081 = BOT2487 = ATCC 58191, ex-type	*Populus nigra*, bark of dead twig	New Zealand	AY236943	AY236888	AY236917
***N. parvum***	**CPC 26119**	***Citrus sinensis*** **x *Poncirus trifoliata*, trunk**	**Italy**	**MW413878**	**MW419196**	**MW419259**
***N. parvum***	**CPC 26120**	***Citrus sinensis*** **x *Poncirus trifoliata*, trunk**	**Italy**	**MW413879**	**MW419197**	**MW419260**
***N. parvum***	**CPC 26121**	***Citrus sinensis*** **x *Poncirus trifoliata*, trunk**	**Italy**	**MW413880**	**MW419198**	**MW419261**
***N. parvum***	**CPC 26122**	***Citrus sinensis*** **x *Poncirus trifoliata*, trunk**	**Italy**	**MW413881**	**MW419199**	**MW419262**
***N. parvum***	**CPC 26124**	***Citrus sinensis*** **x *Poncirus trifoliata*, trunk**	**Italy**	**MW413882**	**MW419200**	**MW419263**
***N. parvum***	**CPC 26126**	***Citrus sinensis*** **x *Poncirus trifoliata*, trunk**	**Italy**	**MW413883**	**MW419201**	**MW419264**
***N. parvum***	**CPC 26127**	***Citrus sinensis*** **x *Poncirus trifoliata*, trunk**	**Italy**	**MW413884**	**MW419202**	**MW419265**
***N. parvum***	**CPC 26128**	***Citrus sinensis*** **x *Poncirus trifoliata*, trunk**	**Italy**	**MW413885**	**MW419203**	**MW419266**
***N. parvum***	**CPC 26129**	***Citrus sinensis*** **x *Poncirus trifoliata*, trunk**	**Italy**	**MW413886**	**MW419204**	**MW419267**
***N. parvum***	**CPC 26130**	***Citrus sinensis*** **x *Poncirus trifoliata*, trunk**	**Italy**	**MW413887**	**MW419205**	**MW419268**
***N. parvum***	**CPC 27866**	***Citrus limon*** **, branch**	**Malta**	**MW413888**	**MW419206**	**MW419269**
***N. parvum***	**CPC 27867**	***Citrus limon*** **, branch**	**Malta**	**MW413889**	**MW419207**	**MW419270**
***N. parvum***	**CPC 27868**	***Citrus limon*** **, branch**	**Malta**	**MW413890**	**MW419208**	**MW419271**
***N. parvum***	**CPC 28173**	***Microcitrus australasica*** **, twig**	**Italy**	**MW413891**	**MW419209**	**MW419272**
***N. parvum***	**CPC 28175**	***Microcitrus australasica*** **, twig**	**Italy**	**MW413892**	**MW419210**	**MW419273**
***N. parvum***	**CPC 28177**	***Microcitrus australasica*** **, twig**	**Italy**	**MW413893**	**MW419211**	**MW419274**
*N. parvum*	CBS 110301 = CAP 074	*Vitis vinifera*	Portugal	AY259098	AY573221	EU673095
*N. parvum*	MFLUCC 15-09002, ex-type of *N. italicum*	*Vitis vinifera*	Italy	KY856755	KY856754	–
*N. parvum*	CBS 137504 = ALG1, ex-type of *N. algeriense*	*Vitis vinifera*, branches	Algeria	KJ657702	KJ657721	–
*N. pennatisporum*	WAC 13153 = MUCC 510, ex-type	*Allocasuarina fraseriana*, healthy stem	Australia: Western Australia	EF591925	EF591976	EF591959
*N. pistaciae*	CBS 595.76, ex-isotype of *Camarosporium pistaciae*	*Pistacia vera*, fruits	Greece	KX464163	KX464676	KX464953
*N. protearum*	CBS 114176 = CPC 1775 = JT 189, ex-type	*Leucadendron salignum* × *L. laureolum* cv. Silvan Red, stems	South Africa	AF452539	KX464720	KX465006
*N. ribis*	CBS 115475 = CMW 7772, ex-type	*Ribes vulgare*	USA	AY236935	AY236877	AY236906
*N. ribis*	CBS 124924 = CMW 28363, ex-type of *N. batangarum*	*Terminalia catappa*, healthy branches	Cameroon	FJ900607	FJ900653	FJ900634
*N. ribis*	CBS 123645 = CMW 14058, ex-type of *N. umdonicola*	*Syzygium cordatum*	South Africa	EU821904	EU821874	EU821844
*N. sinense*	CGMCC3.18315, ex-type	Unknown woody plant	China	KY350148	KY817755	KY350154
*N. sinoeucalypti*	CERC2005 = CGMCC3.18752, ex-type	*Eucalyptus urophylla × E. grandis* tree	China	KX278061	KX278166	KX278270
*N. stellenboschiana*	CBS 110864 = STE-U 4598 = CPC 4598, ex-type	*Vitis vinifera*	South Africa	AY343407	AY343348	KX465047
*N. terminaliae*	CBS 125264 = CMW 26683	*Terminalia sericea*	South Africa	GQ471804	GQ471782	KX465053
*N. ursorum*	CBS 122811 = CMW 24480, ex-type	*Eucalyptus* sp.	South Africa	FJ752746	FJ752709	KX465056
*N. variabile*	CMW 37739, ex-type	*Mimusops caffra*	South Africa	MH558608	–	MH569153
*N. viticlavatum*	CBS 112878 = CPC 5044 = JM 86, ex-type	*Vitis vinifera*	South Africa	AY343381	AY343342	KX465058
*N. vitifusiforme*	CBS 110887 = CPC 5252 = JM5, ex-type	*Vitis vinifera*	South Africa	AY343383	AY343343	KX465061
*N. vitifusiforme*	CBS 120081 = CPC 12925, ex-type of *N. corticosae*	*Eucalyptus corticosa*, leaves	Australia: New South Wales	DQ923533	KX464682	KX464958
*N. vitifusiforme*	CBS 121112 = CPC 5912, ex-type of *N. pruni*	*Prunus salicina*	South Africa	EF445349	EF445391	KX465016
*N. yunnanense*	CSF6142 = CGMCC3.20083, ex-type	*Eucalyptus globulus*, twigs	China	MT028667	MT028833	MT028999

^1^ ATCC: American Type Culture Collection, Virginia, USA; BRIP: Queensland Plant Pathology Herbarium, Brisbane, Australia; CBS: Westerdijk Fungal Biodiversity Institute, Utrecht, the Netherlands; CERC: China Eucalypt Research Centre (CERC), Chinese Academy of Forestry (CAF), China; CGMCC: China General Microbiological Culture Collection Center, Beijing, China; CMM: Culture collection of Phytopathogenic Fungi “Prof. Maria Menezes”, Universidade Federal Rural de Pernambuco, Recife, Brazil; CMW: Tree Pathology Co-operative Program, Forestry and Agricultural Biotechnology Institute, University of Pretoria, South Africa; CPC: Culture collection of Pedro Crous, housed at Westerdijk Fungal Biodiversity Institute; IMI: International Mycological Institute, Kew, U.K.; MFLUCC: Mae Fah Luang University Culture Collection, Chiang Ria, Thailand; MUCC: Murdoch University, Perth, Western Australia; URM: Culture collection Prof. Maria Auxiliadora Cavalcanti, Recife, Brazil. For other codes see the GenBank accession numbers. ^2^ ITS: internal transcribed spacers and intervening 5.8S nrDNA; *TEF1*: partial translation elongation factor 1-alpha gene; *TUB2*: partial β-tubulin gene.

## Supplementary Materials

The following are available online at https://www.mdpi.com/2223-7747/10/3/492/s1, Tables S1–S3. Kruskal-Wallis test results with multiple comparisons for disease severity between different *Botryosphaeriaceae* spp. on artificially inoculated twigs of *C*. *sinensis* (Table S1), *C*. *limon* (Table S2) and *C*. *reticulata* (Table S3).
