# Supplementary material for: Genetic Diversity and Pathogenicity of Botryosphaeriaceae Species Associated with Symptomatic Citrus Plants in Europe
Source: Plants (Basel). 2021 Mar 5;10(3):492. doi: 10.3390/plants10030492 (PMC7999779; doi:10.3390/plants10030492)
Supplement: Supplementary file 1 [file plants-10-00492-s001.pdf]

Supplementary Table S1–S3. Kruskal-Wallis test results with multiple comparisons for disease severity between different *Botryosphaeriaceae* spp. on artificially inoculated twigs of *C. sinensis* (S1), *C. limon* (S2) and *C. reticulata* (S3).

Table S1.

Pairwise comparison between cultivar	Significance *P value**
Dothiorella viticola:Diplodia insularis	0.218
Dothiorella viticola:Diplodia mutila	0.055
Dothiorella viticola:Neofusicoccum luteum	**0.000**
Dothiorella viticola:Diplodia seriata	**0.000**
Dothiorella viticola:Diplodia olivarum	**0.000**
Dothiorella viticola:Lasiodiplodia theobromae	**0.000**
Dothiorella viticola:Neofusicoccum parvum	**0.000**
Dothiorella viticola:Neofusicoccum mediterraneum	**0.000**
Diplodia insularis:Diplodia mutila	0.494
Diplodia insularis:Neofusicoccum luteum	**0.000**
Diplodia insularis:Diplodia seriata	**0.000**
Diplodia insularis:Diplodia olivarum	**0.000**
Diplodia insularis:Lasiodiplodia theobromae	**0.000**
Diplodia insularis:Neofusicoccum parvum	**0.000**
Diplodia insularis:Neofusicoccum mediterraneum	**0.000**
Diplodia mutila:Neofusicoccum luteum	**0.000**
Diplodia mutila:Diplodia seriata	**0.000**
Diplodia mutila:Diplodia olivarum	**0.000**
Diplodia mutila:Lasiodiplodia theobromae	**0.000**
Diplodia mutila:Neofusicoccum parvum	**0.000**
Diplodia mutila:Neofusicoccum mediterraneum	**0.000**
Neofusicoccum luteum:Diplodia olivarum	0.400
Neofusicoccum luteum:Diplodia seriata	0.400
Neofusicoccum luteum:Lasiodiplodia theobromae	0.354
Neofusicoccum luteum:Neofusicoccum parvum	0.191
Neofusicoccum luteum:Neofusicoccum mediterraneum	**0.045**
Diplodia olivarum:Lasiodiplodia theobromae	0.932
Diplodia olivarum:Neofusicoccum parvum	0.642
Diplodia olivarum:Diplodia olivarum	1.000
Diplodia seriata:Lasiodiplodia theobromae	0.932
Diplodia seriata:Neofusicoccum parvum	0.642
Diplodia seriata:Neofusicoccum mediterraneum	0.243
Diplodia olivarum:Neofusicoccum mediterraneum	0.243

Lasiodiplodia theobromae:Neofusicoccum parvum	0.704
Lasiodiplodia theobromae:Neofusicoccum mediterraneum	0.279
Neofusicoccum parvum:Neofusicoccum mediterraneum	0.483

Table S2.

**Pairwise comparison between cultivar**	**Significance *P value****
Dothiorella viticola:Diplodia insularis	0.325
Dothiorella viticola:Diplodia mutila	0.170
Dothiorella viticola:Diplodia seriata	**0.000**
Dothiorella viticola:Neofusicoccum luteum	**0.000**
Dothiorella viticola:Diplodia olivarum	**0.000**
Dothiorella viticola:Neofusicoccum mediterraneum	**0.000**
Dothiorella viticola:Neofusicoccum parvum	**0.000**
Dothiorella viticola:Lasiodiplodia theobromae	**0.000**
Diplodia insularis:Diplodia mutila	0.697
Diplodia insularis:Diplodia seriata	**0.003**
Diplodia insularis:Neofusicoccum luteum	**0.000**
Diplodia insularis:Diplodia olivarum	**0.000**
Diplodia insularis:Neofusicoccum mediterraneum	**0.000**
Diplodia insularis:Neofusicoccum parvum	**0.000**
Diplodia insularis:Lasiodiplodia theobromae	**0.000**
Diplodia mutila:Diplodia seriata	**0.010**
Diplodia mutila:Neofusicoccum luteum	**0.000**
Diplodia mutila:Diplodia olivarum	**0.000**
Diplodia mutila:Neofusicoccum mediterraneum	**0.000**
Diplodia mutila:Neofusicoccum parvum	**0.000**
Diplodia mutila:Lasiodiplodia theobromae	**0.000**
Diplodia seriata:Neofusicoccum luteum	**0.007**
Diplodia seriata:Diplodia olivarum	**0.005**
Diplodia seriata:Neofusicoccum mediterraneum	**0.000**
Diplodia seriata:Neofusicoccum parvum	**0.000**
Diplodia seriata:Lasiodiplodia theobromae	**0.000**
Neofusicoccum luteum:Diplodia olivarum	0.927
Neofusicoccum luteum:Neofusicoccum mediterraneum	0.279
Neofusicoccum luteum:Neofusicoccum parvum	**0.005**
Neofusicoccum luteum:Lasiodiplodia theobromae	**0.000**
Diplodia olivarum:Neofusicoccum mediterraneum	0.322
Diplodia olivarum:Neofusicoccum parvum	**0.006**

Diplodia olivarum:Lasiodiplodia theobromae	**0.000**
Neofusicoccum mediterraneum:Neofusicoccum parvum	0.079
Neofusicoccum mediterraneum:Lasiodiplodia theobromae	**0.005**
Neofusicoccum parvum:Lasiodiplodia theobromae	0.303

Table S3.

**Pairwise comparison between cultivar**	**Significance *P value****
Diplodia mutila:Dothiorella viticola	0.939
Diplodia mutila:Diplodia insularis	0.271
Diplodia mutila:Neofusicoccum luteum	**0.000**
Diplodia mutila:Diplodia seriata	**0.000**
Diplodia mutila:Neofusicoccum parvum	**0.000**
Diplodia mutila:Diplodia olivarum	**0.000**
Diplodia mutila:Lasiodiplodia theobromae	**0.000**
Diplodia mutila:Neofusicoccum mediterraneum	**0.000**
Dothiorella viticola:Diplodia insularis	0.305
Dothiorella viticola:Neofusicoccum luteum	**0.000**
Dothiorella viticola:Diplodia seriata	**0.000**
Dothiorella viticola:Neofusicoccum parvum	**0.000**
Dothiorella viticola:Diplodia olivarum	**0.000**
Dothiorella viticola:Lasiodiplodia theobromae	**0.000**
Dothiorella viticola:Neofusicoccum mediterraneum	**0.000**
Diplodia insularis:Neofusicoccum luteum	**0.000**
Diplodia insularis:Diplodia seriata	**0.000**
Diplodia insularis:Neofusicoccum parvum	**0.000**
Diplodia insularis:Diplodia olivarum	**0.000**
Diplodia insularis:Lasiodiplodia theobromae	**0.000**
Diplodia insularis:Neofusicoccum mediterraneum	**0.000**
Neofusicoccum luteum:Diplodia seriata	**0.031**
Neofusicoccum luteum:Neofusicoccum parvum	**0.021**
Neofusicoccum luteum:Diplodia olivarum	**0.019**
Neofusicoccum luteum:Lasiodiplodia theobromae	**0.001**
Neofusicoccum luteum:Neofusicoccum mediterraneum	**0.000**
Diplodia seriata:Neofusicoccum parvum	0.876
Diplodia seriata:Diplodia olivarum	0.850
Diplodia seriata:Lasiodiplodia theobromae	0.261
Diplodia seriata:Neofusicoccum mediterraneum	0.129
Neofusicoccum parvum:Diplodia olivarum	0.974

Neofusicoccum parvum:Lasiodiplodia theobromae	0.334
Neofusicoccum parvum:Neofusicoccum mediterraneum	0.173
Diplodia olivarum:Lasiodiplodia theobromae	0.350
Diplodia olivarum:Neofusicoccum mediterraneum	0.184
Lasiodiplodia theobromae:Neofusicoccum mediterraneum	0.692
